# Emergency Control Strategies for *Hylurgops longipillus* R. (Coleoptera, Scolytinae) Outbreaks: From Chemical Communication to Integrated Management

**DOI:** 10.3390/insects17080860

**Published:** 2026-08-18

**Authors:** Huanwen Chen, Dan Xie, Li Liu, Lihong Jiang, Xiaowei Chen, Kai Ding, Xinhe Yu, Jia Yu, Defu Chi

**Affiliations:** 1 Key Laboratory for Sustainable Forest Ecosystem Management of Ministry of Education, College of Forestry, Northeast Forestry University, Harbin 150040, China; chenhuanwen2020@126.com (H.C.); xiedan0807@126.com (D.X.); 18347099397@163.com (L.L.); jianglihong199@126.com (L.J.); hcoeng5@163.com (X.C.); jlbssczzxcb@126.com (X.Y.); 2 Northeast Forestry University Maoershan Academiccenter, Harbin 150040, China; dingkai.666@163.com

**Keywords:** *Pinus koraiensis*, chemical ecology, attractant, integrated pest management (IPM), monitoring

## Abstract

Under climate warming, bark beetle outbreaks increasingly threaten forest health. *Hylurgops longipillus*, a native bark beetle in Northeast China, causes patchy mortality in *Pinus koraiensis* plantations. We developed an integrated emergency control system based on host selection chemical ecology. Damage characterization revealed that adults concentrated on trees with 10–50% crown needle yellowing, and infestations expanded as discrete patches radiating from multiple epicenters. Electrophysiological and behavioral assays of host volatiles and some of their isomers yielded a highly effective attractant (69.4 adults per five-trap set over 5 d, 1.8× control) and a repellent reducing ethanol-baited trap catches by 83.3%. Emergency control techniques consisted of integrated management combining removal and safe disposal of infested trees, chemical stump sealing, push–pull trapping, and tree vigor enhancement. Three-year monitoring showed ~94% decline in trap catches, no subsequent outbreaks, and stable control. This attractant-centered, chemical ecology-based system can rapidly suppress *H. longipillus* outbreaks and achieve long-term management, providing a scientific basis and operational framework for similar localized bark beetle emergencies under climate warming.

## Simple Summary

A bark beetle named *Hylurgops longipillus* damages Korean pine plantations in Northeast China, killing trees in scattered patches. To fight these outbreaks, we created an emergency control system based on the beetle’s attraction to tree odors. We observed that the beetles prefer pines with slightly yellow needles and that infestations expand outward from hotspots. Using the natural scents of pine, we developed a powerful attractant that captured an average of 69.4 beetles per set of five traps over five days (1.8 times the standard lure), and a repellent that reduced trap catches by 83.3%. We then combined the removal and safe disposal of infested trees, chemical sealing of stumps, the joint use of the attractant and repellent (a push–pull strategy), and tree vigor enhancement for emergency control of *H. longipillus*. After three years of monitoring, beetle trap catches dropped by about 94%, and no new outbreaks occurred.

## 1. Introduction

Outbreaks of native pests, once considered stable or predictable, are now being driven by the combined pressures of global climate change and human activities [1,2]. In the forest region of Northeast China, for example, *Pinus koraiensis* possesses both excellent ecological service functions and immense economic value [3,4]. Specifically, its ecological services are mainly manifested in sustaining regional ecosystem stability and biodiversity, whereas its economic value is derived primarily from timber, pine nuts, other forest products, and the development of ecotourism. It is the dominant species of the climax forest community in this region [5,6], yet it has long been threatened by a variety of wood-boring pests [7,8]. Notably, external wounds on *P. koraiensis* caused by management activities such as cone harvesting render the trees more susceptible to infestation by secondary bark beetles. *Hylurgops longipillus* Reitter (Coleoptera: Curculionidae: Scolytinae) is a typical wood-boring secondary pest, mainly damaging coniferous trees such as *P. koraiensis* and *P. armandii* [9]. The adults and larvae of *H. longipillus* bore into the phloem of *P. koraiensis* trunks, destroy vascular tissues, cause bark peeling, and in severe cases lead to tree mortality, resulting in ecological and economic losses.

Large-scale outbreaks of bark beetles worldwide have become a major disturbance factor causing dramatic shifts in forest structure, and their impacts are intensifying under climate change [10]. Many of these pests are secondary species, and their outbreaks are typically triggered by external biotic or abiotic stress factors, such as drought and storms [11]. For example, the European spruce bark beetle, *Ips typographus* Linnaeus, has caused widespread spruce mortality in Europe and poses a persistent threat to its temperate and boreal forests [12,13]; the red turpentine beetle, *Dendroctonus valens* LeConte, is a secondary pest in its native range of North America, but after invading China, it has caused severe damage to hundreds of thousands of hectares of forest [14]. Bark beetles and their symbiotic microorganisms cooperatively damage their hosts through interactions where the microorganisms release specific chemical signals that attract the beetles to aggregate and rapidly weaken and kill the host [15,16]. On this basis, researchers have developed a variety of control and monitoring methods targeting bark beetles, including plant-derived attractants and pheromone-based attractants [17,18].

The management of bark beetles is a multi-layered, integrated process, typically encompassing prevention, monitoring, and suppression. At the prevention level, optimizing forest management remains the core approach, such as establishing mixed-species forests [19,20]. At the monitoring level, pheromone traps and trap trees have traditionally been used to monitor population dynamics and achieve local population suppression, while the development of remote identification systems has also provided new technologies for monitoring [21,22,23]. At the suppression level, in addition to sanitation cutting (i.e., removal of infested trees), physical removal, and chemical control [24,25,26]. Early and precise control is critically important for bark beetle pests, especially for species with irregular outbreaks, for which prompt emergency intervention at the early stage represents a critical juncture for population suppression and offers early protection against more extensive outbreak damage.

Integrated Pest Management (IPM) emphasizes the synergy of multiple techniques, in which the “push-pull” strategy, combining attractants and repellents, has become an important direction for the sustainable control of bark beetles. However, relying solely on behavioral manipulation is insufficient for sustained population suppression; it must be integrated with suppression measures, such as the removal of infested trees, to form a complete IPM system [27]. Currently, research on the occurrence patterns and chemical ecology mechanisms of *H. longipillus* is very limited, and there is a lack of targeted monitoring and control techniques. Therefore, systematically elucidating its population dynamics and key chemical ecology mechanisms is a prerequisite for developing specific trapping techniques and achieving effective monitoring and early warning. It is also the scientific basis for incorporating it into an IPM system centered on the “push-pull” strategy and for achieving long-term sustainable control. This study aims to advance knowledge in this direction and provide core theoretical and technical support for building an integrated management strategy against *H. longipillus*.

Focusing on the infestation of *H. longipillus*, this study conducted the following work: (1) Through the use of volatile compounds as mediators, the relationship between the host selection process of *H. longipillus* and the damage severity of *P. koraiensis* was elucidated; (2) the dynamic changes in the activities of terpene synthase (TPS), catalase (CAT), and peroxidase (POD) during the mortality process of *P. koraiensis* infested by *H. longipillus* were analyzed, revealing the decline characteristics of its defense system; (3) by utilizing the attraction and avoidance responses of *H. longipillus* to volatiles, highly effective attractants and repellents were screened; (4) integrated control and long-term monitoring of *H. longipillus* were carried out in the infested areas.

## 2. Materials and Methods

### 2.1. Study Site Overview and Damage Severity Classification of P. koraiensis

Since June 2020, monitoring of damage caused by *H. longipillus* has been conducted in a pure plantation of *P. koraiensis* in Shangzhi City, Heilongjiang Province, China (127.53° E, 45.38° N), and subsequent research on its occurrence patterns has been carried out. The infested stand of *P. koraiensis* had an average tree age of 32 years, an average diameter at breast height of 20.4 cm, and an average tree height of 14.5 m. From May to October 2021, given that the infestation was at an early stage, emergency control measures were applied to all concentrated infestation patches identified within the study plantation, aiming to integrate practical pest management with the ongoing experimental research. Subsequently, from May 2022 to August 2025, the survey scope was expanded to a 4 ha area surrounding the original infestation patches, and an attractant was used to conduct long-term monitoring of *H. longipillus* occurrence while recording changes in the number of damaged trees.

In this study, the damage severity of *P. koraiensis* in areas infested by *H. longipillus* was classified into five classes based on crown and needle appearance symptoms: Class I—healthy trees, which grow vigorously, with normal needle color and no visible damage symptoms; Class II—lightly damaged trees, in which needles show sporadic discoloration (≤10%), indicating the onset of infestation; Class III—moderately damaged trees, in which the proportion of discolored needles increases significantly (approximately 10–50%), indicating progressive damage; Class IV—severely damaged trees, in which the majority of needles (approximately 50–75%) are discolored, and obvious frass deposits are visible on the trunk bark surface; Class V—dead trees, in which all needles are yellowed and withered but have not yet fallen off extensively, indicating that the trees have died.

### 2.2. Sample Collection and Preparation

With a sterile scalpel, a piece of phloem approximately 8 cm × 8 cm (including part of the cambium) was excised from the trunk of each sample tree at 1.0–1.5 m above ground level, placed into a sterile sampling bag, kept on ice, and quickly transported back to the laboratory.

The samples were divided into two portions: one portion was cut into small pieces (2 cm × 1 cm), sealed, and stored at −20 °C for behavioral tests (and were warmed to room temperature before testing); the remainder was flash-frozen in liquid nitrogen and ground into a fine powder. A 2.5 g portion of the powder was heated at 105 °C for 3 min to inactivate enzymes, then dried at 60 °C to constant weight, ground again, and passed through a 40-mesh sieve to obtain the final dried sample for enzyme activity assays.

### 2.3. Volatile Composition and Biochemical Analysis

Volatile sampling was conducted using a Silonite™ air sampling canister (ENTECH, Simi Valley, CA, USA). The sampling site was the trunk of *P. koraiensis* at 1.5 m above ground level. The collected air samples were subjected to cryo-focusing and thermal desorption using a preconcentrator (ENTECH 7100A, Simi Valley, CA, USA). Volatile compounds were analyzed using a gas chromatography–mass spectrometry (GC-MS) system (Agilent Technologies, Santa Clara, CA, USA), consisting of a 6890N gas chromatograph coupled to a 5975C mass selective detector. A DB-5 fused-silica capillary column (30 m × 0.25 mm × 0.25 μm) was used, with high-purity helium as the carrier gas. The oven temperature program was as follows: initial temperature 40 °C, increased at 5 °C/min to 90 °C, then at 8 °C/min to 250 °C, and held for 25 min. Mass spectra were acquired in electron impact ionization mode (70 eV) with an ion source temperature of 230 °C and a mass scan range of *m/z* 50–500. Compounds were tentatively identified by matching their mass spectra with those in standard mass spectral libraries.

All enzyme activity assay kits were purchased from Suzhou Grace Biotechnology Co., Ltd., Suzhou, China. The measured indicators included catalase (CAT), peroxidase (POD), and terpene synthase (TPS). Enzyme activity assays were performed strictly according to the manufacturer’s instructions. Three technical replicates were performed for each independent sample, and the arithmetic mean was taken as the final result to minimize random errors.

### 2.4. Chemical Reagents, Pesticides, and Tree Nutrient Supplements Used in the Experiments

Information on the chemical reagents used in the experiments is as follows. The purity values indicated in parentheses (e.g., ≥98%) represent the supplier’s guaranteed minimum purity as stated in the commercial product specifications, rather than the certified purity of specific batches. All compounds were stored under the conditions recommended by the suppliers and were freshly prepared immediately before each behavioral assay to minimize any potential degradation. The reagents were: α-pinene (CAS#80-56-8, ≥98%, Aladdin, Shanghai, China), (+)-α-pinene (CAS#7785-70-8, ≥98%, Aladdin), (-)-α-pinene (CAS#7785-26-4, ≥98%, Aladdin), β-pinene (CAS#127-91-3, ≥95%, Aladdin), (-)-β-pinene (CAS#18172-67-3, ≥98%, Aladdin), γ-terpinene (CAS#99-85-4, ≥95%, Macklin, Shanghai, China), 3-carene (CAS#13466-78-9, ≥96%, Macklin), α-phellandrene (CAS#99-83-2, ≥85%, Macklin), camphene (CAS#79-92-5, ≥95%, Macklin), myrcene (CAS#123-35-3, ≥92%, Macklin), α-terpinene (CAS#99-86-5, ≥95%, Macklin), limonene (CAS#138-86-3, ≥95%, Macklin), (+)-limonene (CAS#5989-27-5, ≥95%, Macklin), (-)-limonene (CAS#5989-54-8, ≥95%, Macklin), D-camphor (CAS#464-49-3, ≥96%, Macklin), bornyl acetate (CAS#76-49-3, ≥97%, Macklin), (+)-longifolene (CAS#475-20-74, ≥95%, Macklin), ethanol (CAS#64-17-5, ≥99.5%, Macklin).

Dinotefuran, an insecticide used for root drenching, was purchased from Heilongjiang Pingshan Forestry Pharmaceutical Factory, Harbin, China. The tree nutrient solution was purchased from Harbin Sanshan Forestry Pharmaceutical Factory, Harbin, China.

### 2.5. Electrophysiological and Behavioral Responses of H. longipillus to Authentic Volatile Standards

The electroantennographic (EAG) responses of antennae to volatiles were recorded as follows. Test volatiles were dissolved in liquid paraffin and prepared into five concentrations: 0.01, 0.1, 1, 10, and 100 μg/μL. For each stimulation, 10 μL of the test solution was applied to a filter paper strip (10 mm × 50 mm), and a 300 mL/min airstream (duration 0.5 s, inter-stimulus interval 30 s) was directed over the antenna. Each concentration was tested five times, and liquid paraffin was used as the negative control (once before and once after the stimuli). Each antenna was used for no longer than 20 min. For each volatile, five females and five males were tested, with concentrations presented in ascending order. After each volatile was tested, the Pasteur pipette and connecting tubing were replaced.

**Figure 1:**
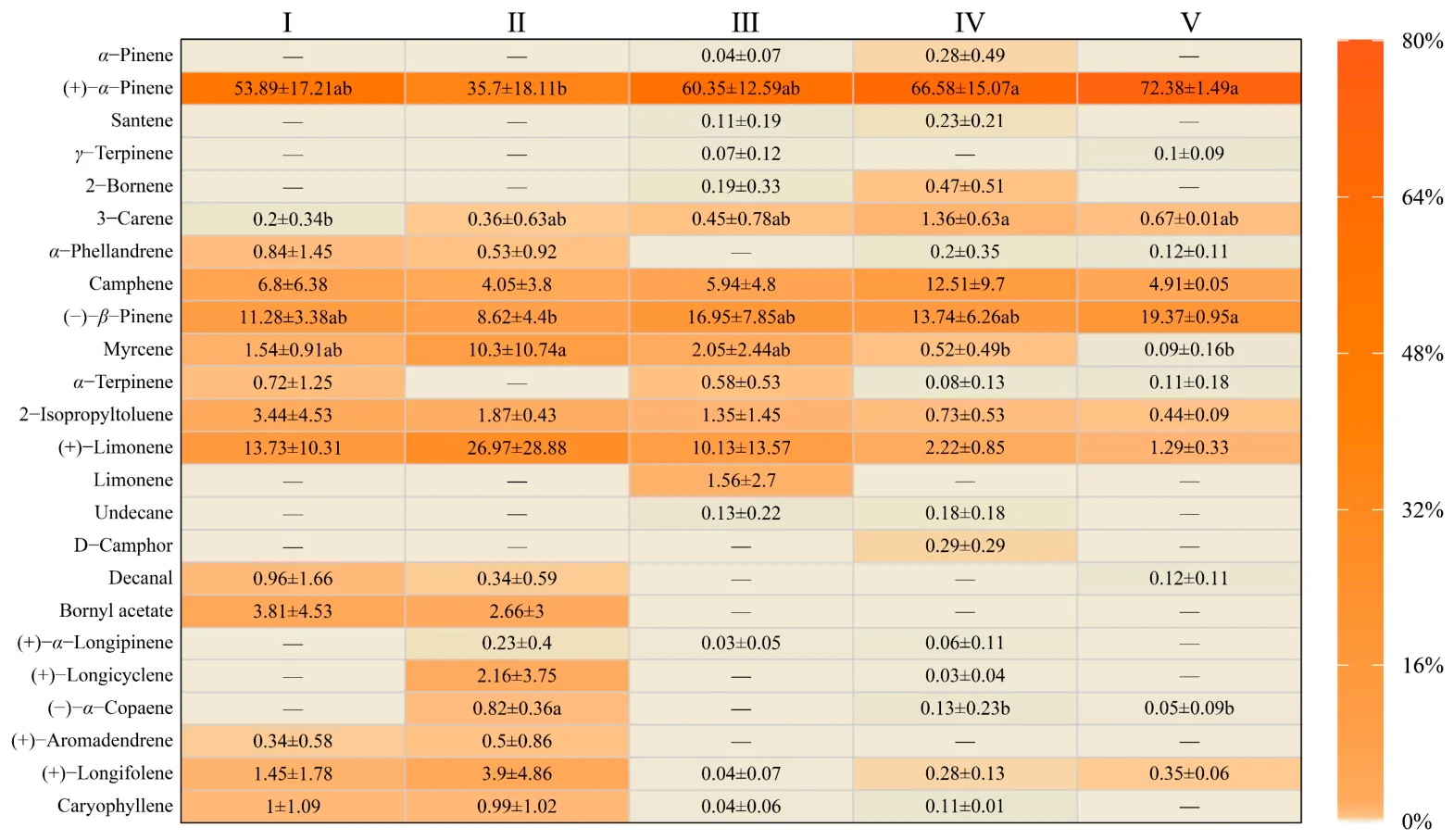
Composition and relative content of volatiles from the trunk phloem of *P. koraiensis* under different health conditions. The color gradient from light yellow to dark red indicates increasing relative content; “—” indicates that the compound was not detected; numbers within boxes represent mean ± SD of relative content. Different lowercase letters in the same row denote significant differences in the relative content of the same volatile among phloem samples of *P. koraiensis* at different damage severities (one-way ANOVA, Tukey’s HSD post hoc test, *p* < 0.05).

The preference of *H. longipillus* adults for the phloem of *P. koraiensis* was tested as follows. The olfactory behavioral assay apparatus (Figure 1) was used to test the host selection preference. The main body of the apparatus was equipped with six traps, and beneath each trap, a polypropylene tube with dense ventilation holes was connected to hold the odor source. An air pump connected to the top cover drew air at a constant total flow rate (2000 sccm) evenly from each choice arm, directing the odor from bottom to top into the central choice arena and establishing a stable directional airflow field. The experimental setup included five treatment groups (corresponding to phloem samples from damage severity classes I–V) and one blank control group (containing wood chips of *P. koraiensis* that had been degreased and re-equilibrated to ambient humidity, to control for the potential influence of ambient moisture on selection). At the start of the test, the adults were released into the central choice arena and allowed to move freely; the number of adults falling into each trap within 300 s was recorded. Each treatment was replicated six times, and after each replicate, the apparatus was cleaned with absolute ethanol and dried, and the positions of the odor source tubes were rotated clockwise to eliminate any positional bias.

Y-tube olfactometer bioassays were conducted as follows. At 25 ± 1 °C, 10 μL of the test solution was applied to a filter paper strip, and the strip was placed in one arm of the Y-tube. An equal amount of liquid paraffin was applied to another filter paper strip and placed in the other arm as the control. After both arms were ventilated with an airflow of 0.5 L/min for 15 s, the test insects were introduced. An insect was recorded as having made a choice if it entered one arm and moved more than one-third of the arm length within 1 min. After every five insects, the positions of the two arms were exchanged, and after every ten insects, the apparatus was cleaned with 95% ethanol and dried at 180 °C. For each group, ten females and ten males were tested, and the experiment was replicated three times. When testing behavioral disrupting agents, the slow-release bag (wall thickness 0.25 mm; Shenzhen Prince New Materials Co., Ltd., Shenzhen, China) was directly placed into one arm as the odor source, while the other arm served as a control.

Wind tunnel bioassays were conducted as follows. A push-type horizontal wind tunnel (2.2 m long, cross section 0.5 m × 0.5 m) was used under conditions of 25 ± 1 °C and 60 ± 10% relative humidity (RH). Odor sources (volatile blends or disrupting agents) were loaded into slow-release dispensers and placed at the upwind end of the tunnel. Along the direction of the airflow, the tunnel was divided into a near-source zone (0.6 m), a middle zone (1.0 m), and a far-source zone (0.6 m). Thirty adults of *H. longipillus* were released at the center of the wind tunnel, and the number of individuals entering each zone within 20 min was recorded, and crawling behavior was observed. Each treatment was replicated five times. For each replicate, a new group of adults was used, and the same individuals were not reused.

### 2.6. Field Trials and Emergency Control

Given that *H. longipillus* damage occurs in patches, all field behavioral disruption trials were conducted within and immediately adjacent to active infestation patches. Cross-vane traps (Hangzhou Pheromone Biotechnology Co., Ltd., Hangzhou, China) were deployed in a grid pattern at 20 m intervals inside each patch (with the collection bucket suspended 1 m above the ground). Behavioral disrupting agents were released from polyethylene slow-release dispensers. For each agent formulation (Table 1), five replicate traps were arranged in a randomized complete block design, with blocks positioned within the patches to control for spatial variability in beetle density. During the trial, all traps were inspected every five days over a 25-day period, and the total number of *H. longipillus* adults captured by the five traps of each formulation was recorded. Data collection was conducted five times in total to evaluate the effectiveness of behavioral disruption.

Infested *P. koraiensis* trees were felled using chainsaws, and the stumps were drenched with 5 L of 1000 mg/L dinotefuran, then covered with 0.2 mm thick plastic film. In the infestation center, baffle traps (lures: attractant formulation II) were deployed. At a distance of 20 m from the center, repellent dispensers (repellent formulation II, without ethanol) were hung. The traps were spaced at 10 m intervals. Meanwhile, a balanced tree nutrient solution mainly composed of compound amino acids, humic acids, and macronutrients was applied to enhance tree vigor. For long-term monitoring, baffle traps were used, with lures identical to attractant formulation II.

### 2.7. Statistical Analysis

Graphs were generated using R version 4.5.2 and GraphPad Prism 10, and all data analyses were performed in R version 4.5.2. For the volatile data, compounds were identified by searching the NIST17 library combined with manual spectral interpretation, and their relative contents were calculated using the peak area normalization method. For continuous data, including the relative content of phloem volatiles, enzyme activities, and EAG values, one-way analysis of variance (ANOVA) was used to test for differences among treatment groups, followed by Tukey’s HSD post hoc multiple comparisons (significance level set at *p* < 0.05). For the preference test of *H. longipillus* adults for the phloem of *P. koraiensis* at different damage severities, a Beta regression model was used, and post hoc multiple comparisons were adjusted by Tukey’s HSD, *p* < 0.05. Data from Y-tube olfactometer and wind tunnel behavioral choice tests were analyzed using chi-square tests (χ^2^ test). To evaluate the efficacy of different behavioral disrupting agents in regulating the behavior of *H. longipillus*, we constructed generalized linear mixed models (GLMMs) using the glmmTMB package, with the number of trapped beetles fitted with a negative binomial distribution. Least-squares means for the treatment variables were obtained using the emmeans package in R, and Tukey-adjusted post hoc pairwise comparisons were performed to identify significant differences in beetle captures among treatment groups.

## 3. Results

### 3.1. Volatile Composition and Changes in Relative Content of the Trunk Phloem of P. koraiensis Across Damage Severity Classes

Through the analysis of volatiles from the phloem of *P. koraiensis* at different damage classes, a total of 24 compounds were identified, mainly including 16 monoterpenes, 5 sesquiterpenes, and 3 non-terpenoid compounds. Their composition and relative content exhibited systematic differences across the damage classes (Figure 1). Further analysis revealed that eight compounds, namely (+)-α-pinene, 3-carene, camphene, (-)-β-pinene, myrcene, 2-isopropyltoluene, (+)-limonene, and (+)-longifolene, were detected in the phloem of *P. koraiensis* trunks across all damage classes, constituting the basal volatile profile of *P. koraiensis*. After being damaged, *P. koraiensis* released specific volatiles, including α-pinene, santene, γ-terpinene, 2-bornene, limonene, undecane, D-camphor, (+)-α-longipinene, (+)-longicyclene, and (-)-α-copaene. Furthermore, α-pinene, santene, 2-bornene, limonene, undecane, and D-camphor were specifically detected in Class III and Class IV damaged trees. In terms of relative content, (+)-α-pinene, (-)-β-pinene, and (+)-limonene were the dominant components, and their dynamics showed a significant pattern: specifically, the relative contents of (+)-α-pinene and (-)-β-pinene exhibited an overall trend of first decreasing and then increasing, reaching their highest levels at the most severe damage stage, with average values of 72.4% and 19.4%, respectively; in contrast, the relative content of (+)-limonene showed a trend of first increasing and then decreasing, peaking at the early damage stage and reaching its lowest level at the most severe damage stage, with average values of 37.0% and 1.3%, respectively.

### 3.2. Host Selection Preference of H. longipillus for P. koraiensis in Relation to Damage Severity

**Figure 2:**
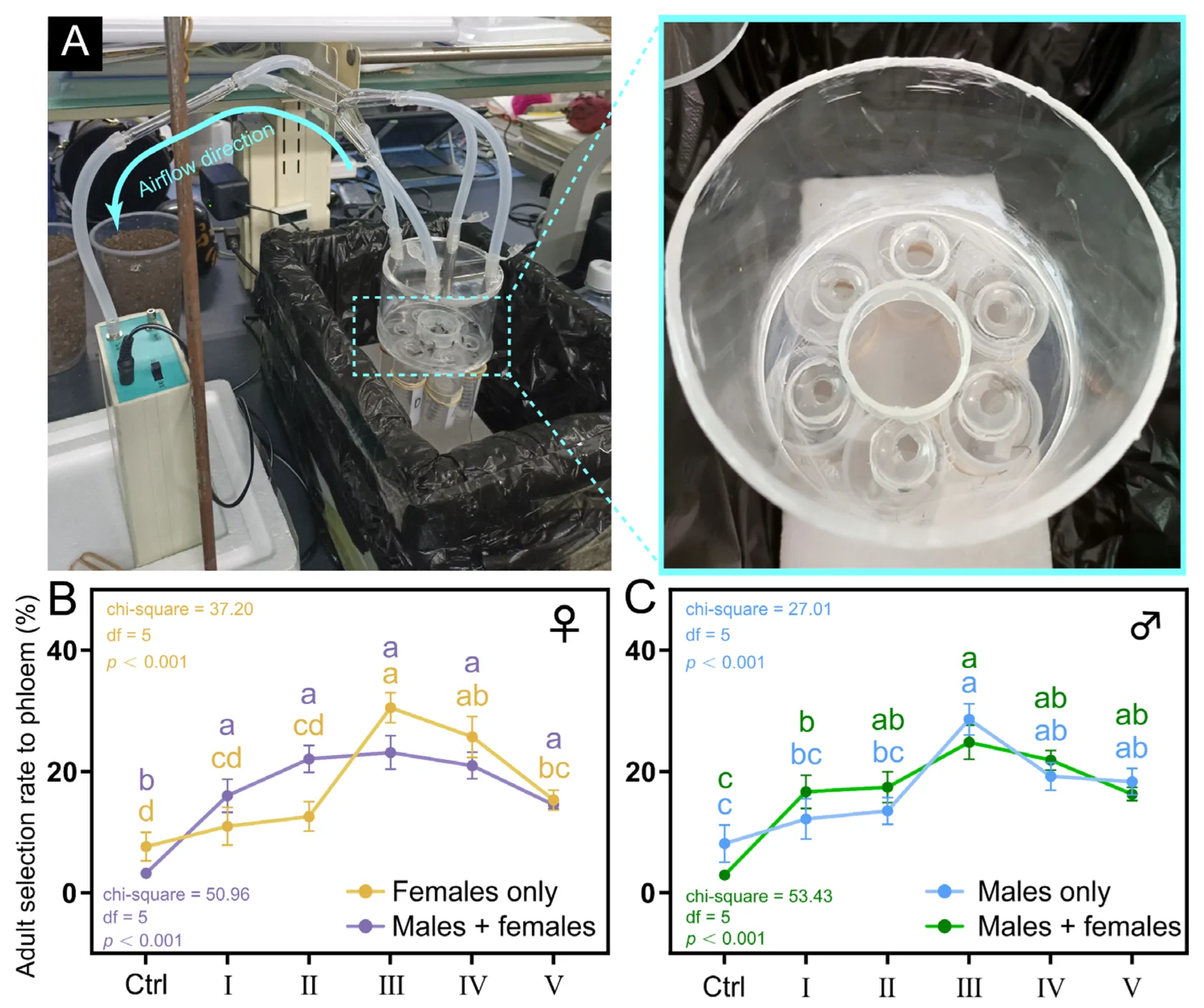
Preference of female and male adults of *H. longipillus* for phloem of *P. koraiensis* across damage severity classes. (**A**) Behavioral choice apparatus for testing the selection behavior of *H. longipillus*. (**B**) Selection rates of female adults for phloem at different damage severities under isolated conditions (only females present) and mixed conditions (males and females tested together). (**C**) Selection rates of male adults for phloem at different damage severities under isolated conditions (only males present) and mixed conditions (males and females tested together). In all tests, the control consisted of phloem from healthy *P. koraiensis* that was degreased and re-moistened; in panels (**B**,**C**), the line graphs compare the selection rates of adults for each phloem type between the two social conditions, and different lowercase letters indicate significant differences in odor selection by the same sex between the two conditions (based on a Beta regression model, post hoc multiple comparisons adjusted by Tukey’s HSD, *p* < 0.05).

The selection behavior of *H. longipillus* toward the phloem of *P. koraiensis* was evaluated using an olfactory choice apparatus (Figure 2A). The results showed that *H. longipillus* exhibited significant differences in preference for phloem from *P. koraiensis* with different damage severities, with phloem from moderately damaged (Class III) trees being the most attractive (Figure 2B). Specifically, when adults of both sexes were tested separately, the selection rates of both sexes for Class III damaged trees were higher than those for the control and other damage classes, reaching 30.55 ± 2.50% and 28.62 ± 2.58%, respectively; however, when adults of both sexes were tested together, although their selection rates for Class III damaged trees remained the highest, they decreased to 23.15 ± 2.76% and 24.84 ± 2.83%, respectively. Compared with the separate tests, the selection rates of females and males for Class I and Class II *P. koraiensis* increased in the mixed test: for Class I damaged trees, the selection rate of females increased by an average of 5.06 percentage points, and that of males increased by 4.47 percentage points; for Class II damaged trees, the increase was 9.51 percentage points for females and 3.93 percentage points for males. In contrast, the selection rates of females and males for the control decreased by 4.38 and 5.22 percentage points, respectively.

### 3.3. Changes in the Activities of Related Enzymes in the Phloem of P. koraiensis During Infestation

**Figure 3:**
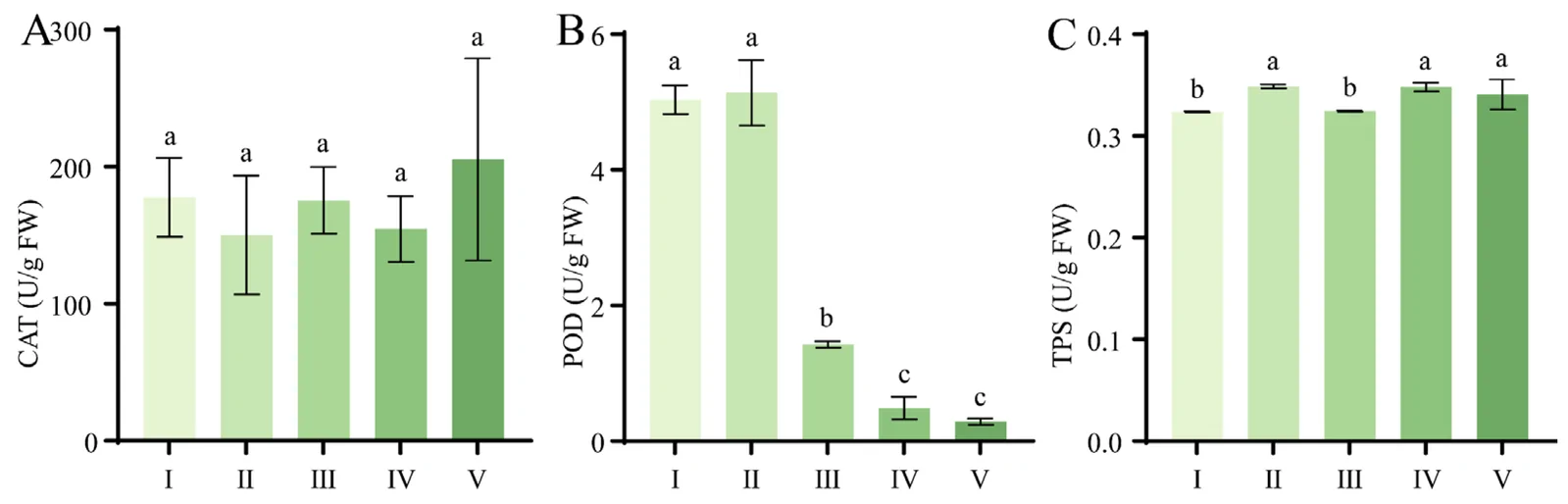
Changes in enzyme activities of *P. koraiensis* under different health conditions. (**A**) CAT activity at different damage severities; (**B**) POD activity in the phloem at different damage severities; (**C**) TPS activity at different damage severities (one-way ANOVA, Tukey’s HSD post hoc test, *p* < 0.05). Different lowercase letters indicate significant differences.

Measurement of enzyme activities in the trunk phloem tissues of *P. koraiensis* at different damage severities showed that, during the progression from *H. longipillus* infestation to tree death, three defense-related enzymes (CAT, POD, and TPS) exhibited impaired responses to varying degrees (Figure 3). Specifically, CAT activity in the phloem of *P. koraiensis* showed no significant difference across all damage classes (F_4,20_ = 1.294, *p* = 0.306), with minimal changes; it neither exhibited a stress-induced increase at the early stage of infestation nor showed compensatory enhancement in the middle and late stages (Figure 3A). POD activity was maintained only briefly before declining sharply as damage severity increased (F_4,20_ = 474.3, *p* < 0.0001), indicating that the defense system of *P. koraiensis* entered an irreversible decline from the early stage of bark beetle infestation (Figure 3B). Although TPS activity increased to some extent after infestation, its overall change was relatively moderate (F_4,20_ = 15.97, *p* < 0.0001), and the trees consistently failed to synthesize sufficient terpenoid defense compounds (Figure 3C). Collectively, these results indicate that *P. koraiensis* has weak resistance to *H. longipillus*, and both its antioxidant and chemical defense systems fail to mount an effective response, leading to the near-collapse of the defense system at an early stage of infestation.

### 3.4. EAG and Behavioral Responses of H. longipillus Adults to Volatiles Associated with the Phloem of P. koraiensis

**Figure 4:**
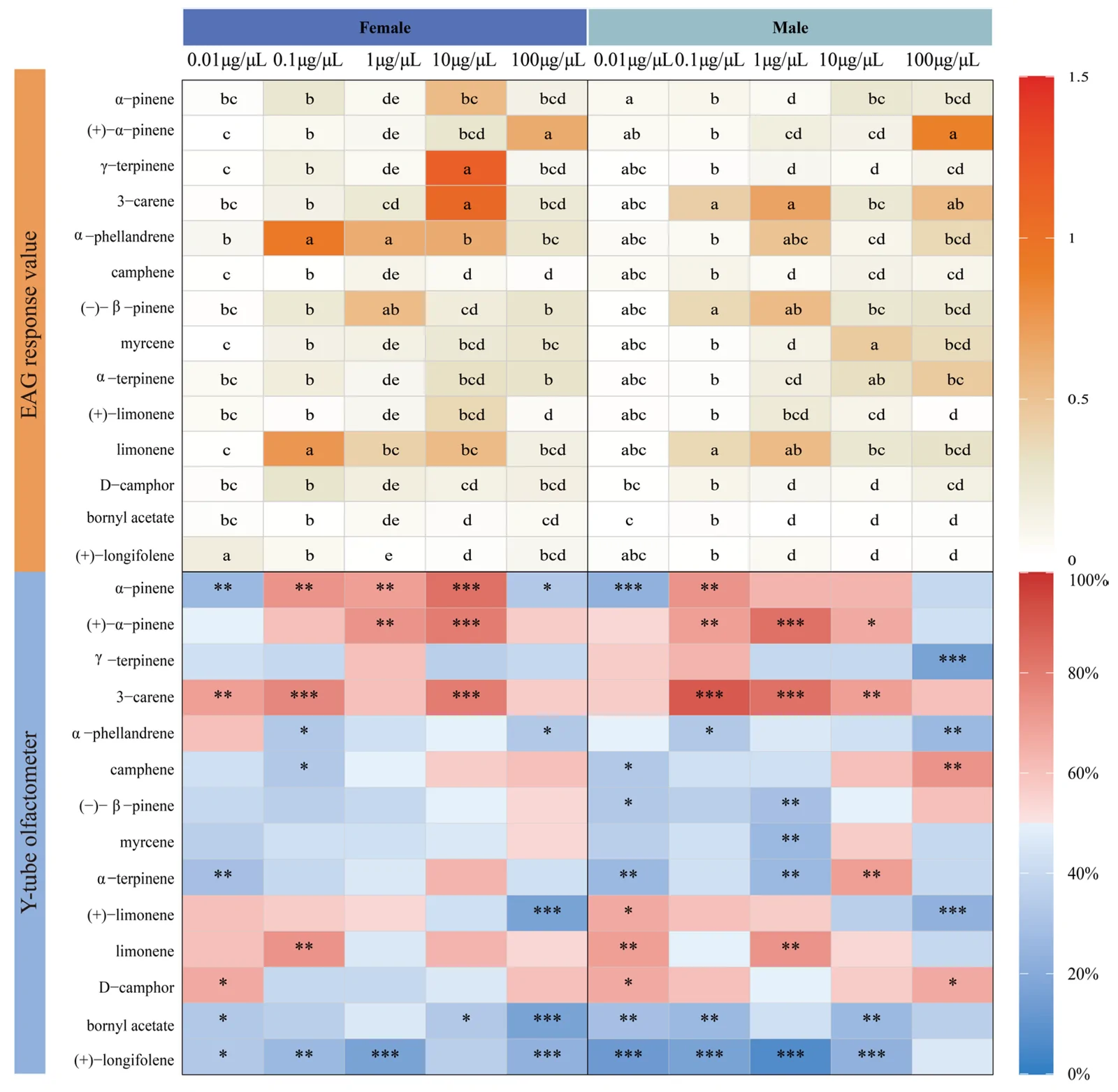
Electroantennographic and behavioral responses of *H. longipillus* to related volatiles. In the EAG response value panel, the color gradient from white to dark red indicates increasing response values; different lowercase letters denote significant differences in the olfactory responses of *H. longipillus* to different volatiles at the same concentration (*p* < 0.05, Tukey’s HSD test, one-way ANOVA, *n* = 5). In the Y-tube olfactometer panel, “*” indicates a significant difference in the selection of *H. longipillus* for odor sources (chi-square test, *p* < 0.05); “**” indicates *p* < 0.01; “***” indicates *p* < 0.001.

EAG was used to investigate the olfactory sensitivity of *H. longipillus* to volatiles from *P. koraiensis* and specific volatiles released after infestation. The results revealed that female adults were generally more sensitive to the volatiles than males, and some volatiles elicited strong EAG responses at relatively low concentrations (Figure 4). Specifically, female adults exhibited significant differences in EAG responses among volatiles at 10 μg/μL (F_13,56_ = 18.84, *p* < 0.0001), whereas male adults showed significant differences at 100 μg/μL (F_13,56_ = 9.69, *p* < 0.0001). In behavioral assays, both female and male adults displayed positive orientation to α-pinene and (+)-α-pinene at 0.1, 1, and 10 μg/μL, and to 3-carene at all tested concentrations. The attractive effects of the remaining compounds were unstable across concentrations, whereas (+)-limonene at 100 μg/μL, and bornyl acetate and (+)-longifolene at all tested concentrations, exhibited relatively strong repellent effects on both sexes.

### 3.5. EAG and Behavioral Responses of H. longipillus to Isomers of Major Volatiles from P. koraiensis

**Figure 5:**
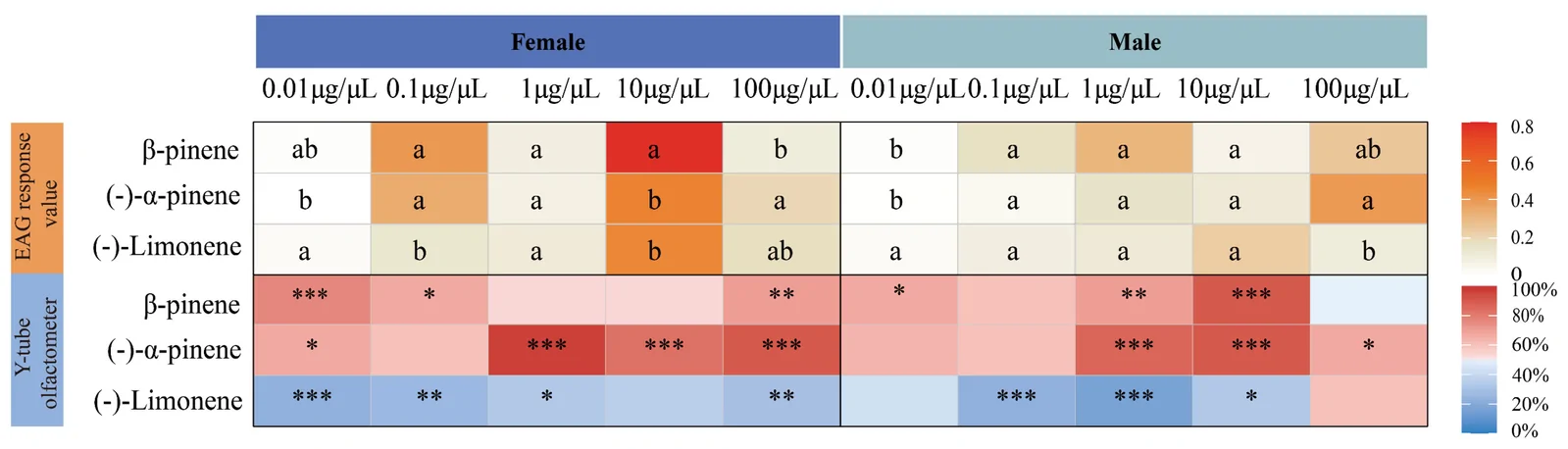
EAG and behavioral responses of female and male adults of *H. longipillus* to isomers of major volatiles from *P. koraiensis*. In the EAG response value panel, the color gradient from white to dark red indicates increasing response values; different lowercase letters denote significant differences in the olfactory responses of *H. longipillus* to different volatiles at the same concentration (*p* < 0.05, Tukey’s HSD test, one-way ANOVA, *n* = 5). In the Y-tube olfactometer panel, “*” indicates a significant difference in the selection of *H. longipillus* for odor sources (chi-square test, *p*< 0.05); “**” indicates *p* < 0.01; “***” indicates *p* < 0.001.

To evaluate the potential of major volatile isomers for use as behavioral disrupting agents, EAG and behavioral assays were conducted to assess the responses of female and male adults of *H. longipillus* to isomers of major volatiles from *P. koraiensis* (Figure 5). The results showed that female adults of *H. longipillus* exhibited the strongest EAG responses to all three candidate volatiles at 10 μg/μL, whereas the EAG responses of male adults did not vary significantly across the tested concentrations. β-pinene and (-)-α-pinene showed significant attraction for both female and male adults of *H. longipillus*, while (+)-limonene showed a significant repellent effect on both sexes. These results indicate that these isomers have the potential to serve as components of behavioral disrupting agents for *H. longipillus*.

### 3.6. Behavioral Responses of H. longipillus to Formulated Attractants and Repellents

**Figure 6:**
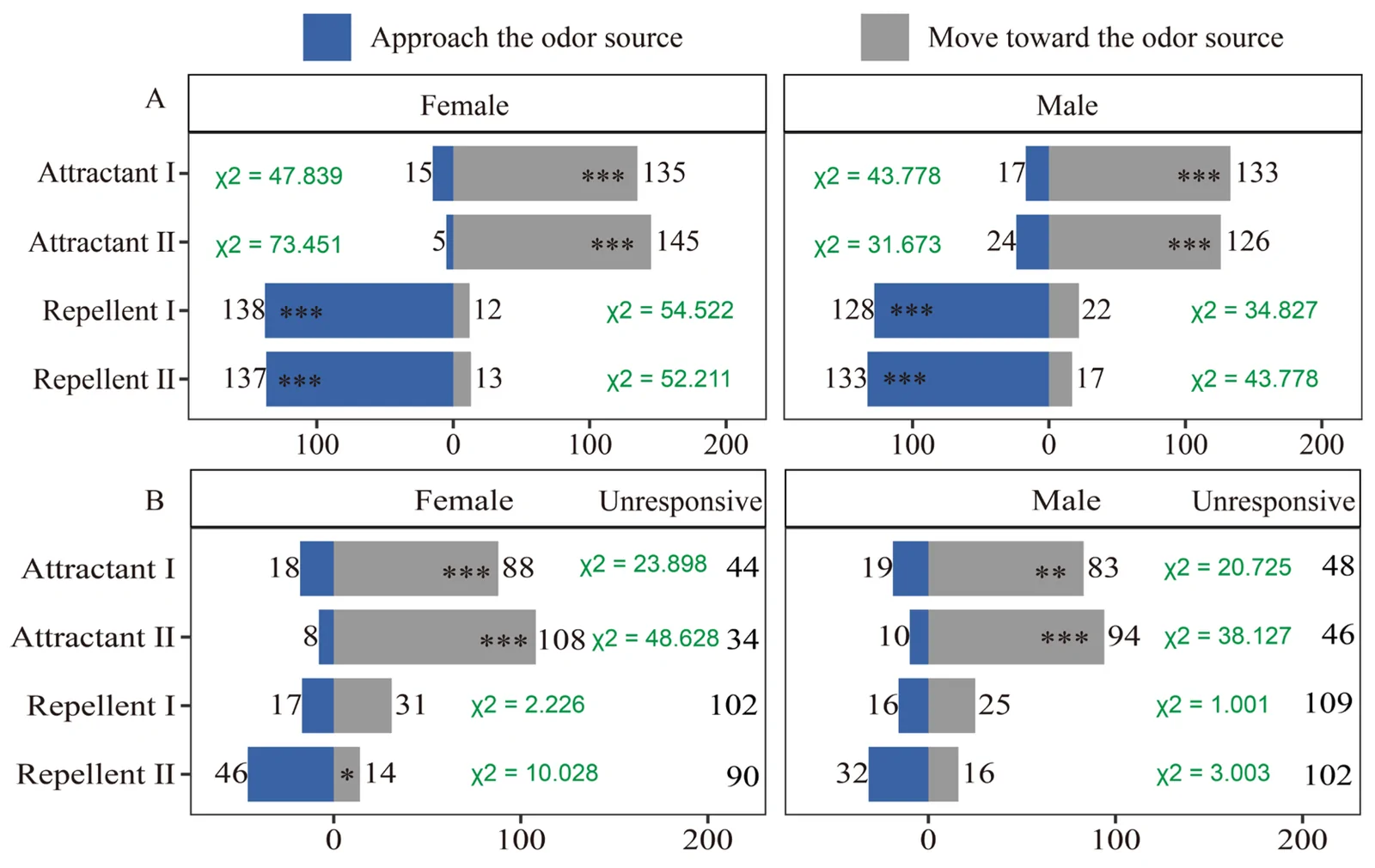
Behavioral responses of *H. longipillus* to four formulated compounds. (**A**) Behavioral responses of both sexes in the Y-tube olfactometer. (**B**) Behavioral responses of both sexes in the wind tunnel. Blue indicates the number of individuals exhibiting negative orientation, and grey indicates the number exhibiting positive orientation (χ^2^ test, *p* values corrected by the Bonferroni method, *p* < 0.05; *n* = 150); “*” indicates *p* < 0.05; “**” indicates *p* < 0.01; “***” indicates *p* < 0.001.

Based on the electrophysiological and behavioral screening results of *H. longipillus* in response to different volatiles, (+)-α-pinene, (-)-α-pinene, α-pinene, β-pinene, and 3-carene were selected as candidate attractant components, while bornyl acetate, (+)-longifolene, and (-)-limonene were selected as candidate repellent components (Figure 4 and Figure 5). On this basis, these compounds were formulated in different combinations (Table 1), encapsulated in slow-release dispensers as odor sources, and further tested for their effects on the behavior of *H. longipillus* through behavioral assays (Figure 6). The results showed that female and male adults exhibited highly consistent response trends to the attractants and repellents. Both attractant formulations showed significant attraction in both the Y-tube olfactometer and wind tunnel experiments. The repellents displayed relatively weak behavioral disruption in the wind tunnel: female adults showed no significant response to Repellent I (χ^2^ = 2.226, *p* = 0.136), and male adults were insensitive to both Repellent I (χ^2^ = 1.001, *p* = 0.317) and Repellent II (χ^2^ = 3.003, *p* = 0.083); the repellents mainly caused a loss of orientation in most adults.

### 3.7. Effects of Behavioral Disrupting Agents and Infested Tree Removal on Trap Catches of H. longipillus

**Figure 7:**
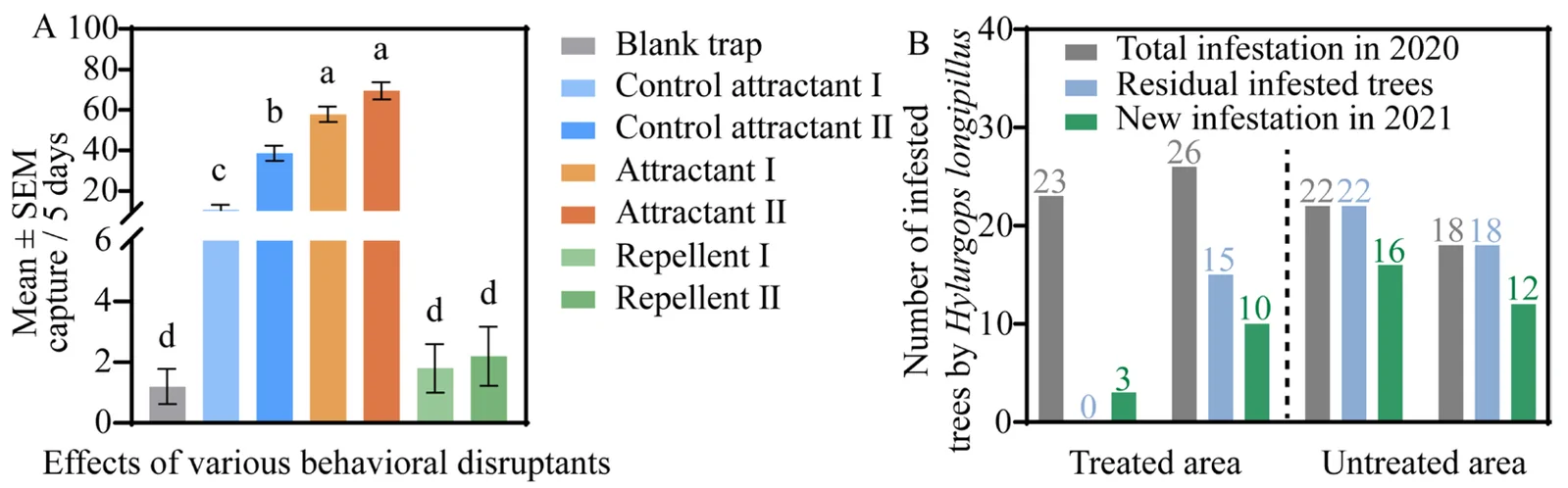
Effects of behavioral disrupting agents on the host selection behavior of *H. longipillus*. (**A**) Effect of formulated behavioral disrupting agents on trap catches of *H. longipillus*. Different lowercase letters indicate significant differences in trap catches among different attractants (GLMMs, *p* < 0.05). (**B**) Effect of infested tree removal on the increase in newly infested trees in the following year.

Compounds that elicited strong responses in *H. longipillus* during laboratory electrophysiological and behavioral tests were formulated, and field trapping experiments were conducted (Figure 7). The results showed that the numbers of beetles attracted by attractant I (estimate = 0.404, z = 3.968, *p* = 0.0014) and attractant II (estimate = 0.587, z = 5.934, *p* < 0.0001) were significantly higher than those attracted by the (+)-α-pinene lure, with mean catches (± SD) of 57.8 ± 3.8 and 69.4 ± 4.2 adults per five traps over five days, respectively; given that ethanol was a common solvent in all formulated lures, ethanol alone was used as the control for the repellent treatments. The numbers of beetles captured by repellent I (estimate = −1.792, z = −4.944, *p* < 0.0001) and repellent II (estimate = −1.792, z = −4.944, *p* < 0.0001) were both significantly lower than those in the control, with mean catches (± SD) of 1.8 ± 0.8 and 2.2 ± 1.0 adults per five traps over five days, respectively (Figure 7A). Four areas infested by *H. longipillus* were selected, and the infested trees within the stands were completely removed, partially removed, or retained. The number of newly infested trees in the following year was recorded (Figure 7B). The results showed that the removal of infested trees significantly reduced the number of newly infested *P. koraiensis* trees in the surrounding area in the following year.

### 3.8. Integrated Control of H. longipillus and Monitoring of Control Efficacy

**Figure 8:**
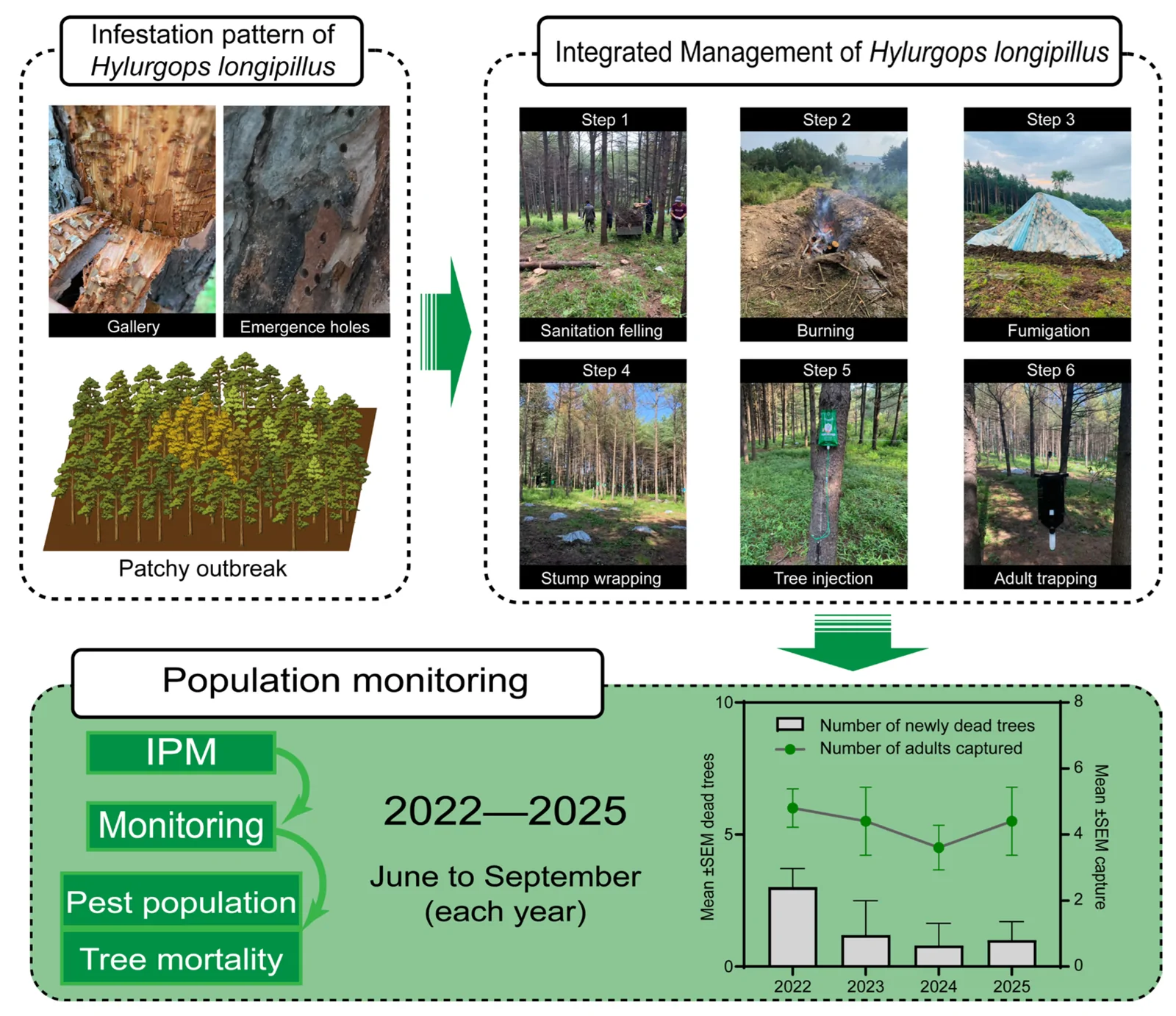
Emergency control and permanent monitoring of *H. longipillus*. The right panel shows adult trap catches (line chart) and the number of newly dead trees (bar chart) recorded in five *H. longipillus* outbreak areas during 2022–2025. In each area, five traps were deployed and adults were collected every five days in July of each year. Surveys of dead trees were conducted in early September annually.

In response to the monitored patchy damage and spread tendency of *H. longipillus*, we conducted systematic emergency control (Figure 8): first, all symptomatic infested trees were thoroughly felled and then incinerated or fumigated to eliminate the core pest sources; subsequently, the stumps were subjected to chemical root drenching and sealing treatment to block pest spread, while traps were densely deployed in the core infested area to trap and kill active adults and reduce the risk of aerial dispersal; in addition, supplemental nutrient solution was applied to nearby healthy *P. koraiensis* at high risk of infestation to enhance their vigor. After three years of continuous monitoring (2022–2025), no new concentrated infestation patches (defined as clusters of ≥5 newly infested trees within a 20 m radius) were detected in the managed area. Beetle captures in monitoring traps remained consistently low (averaging fewer than 5 adults per five trap per inspection), and the number of newly damaged trees did not increase over the monitoring period.

## 4. Discussion

Due to their cryptic and aggregative infestation habits, bark beetles often cause periodic or occasional outbreaks [28,29]; for newly discovered species, or those that have not yet been reported to cause severe damage, effective monitoring and control methods are generally lacking [30]. In the distribution area of *P. koraiensis* in Northeast China, *H. longipillus* primarily attacks the trunk below the crown, exhibiting a patchy outbreak pattern and a tendency to spread to adjacent healthy trees. This study found that after infestation, the antioxidative responses in *P. koraiensis* were not effectively activated, and sufficient amounts of terpenoid defense compounds were not synthesized, leading to the rapid collapse of the host’s defense system. Based on this phenomenon, we conducted research on the chemical ecology mechanisms underlying the infestation of *P. koraiensis* by *H. longipillus*, and, relying on the host selection characteristics of *H. longipillus*, successfully developed a highly effective attractant. Through the implementation of emergency control measures and long-term monitoring, the large-scale damage and spread of *H. longipillus* were effectively controlled. When interpreting these monitoring results, it is important to consider the potential influence of weather conditions, as meteorological factors such as temperature and precipitation can significantly affect the flight activity and trap catches of bark beetles. However, during our monitoring period, weather conditions remained relatively stable without significant fluctuations. Coupled with the observed trend that the number of newly infested trees did not show a notable increase, this further suggests that the consistently low trap catches are unlikely to be an artifact of unfavorable weather, and instead supports the effectiveness of the integrated management measures. The above results indicate that the outbreak damage caused by *H. longipillus* is preventable and controllable, and that the combination of monitoring tools and emergency control strategies can effectively curb the damage caused by this pest in forest areas.

Climate change has triggered outbreaks or elevated outbreak risks of bark beetles in many parts of the world in recent years [31,32]. creating an urgent need to develop effective strategies to cope with such ecological crises. With the *H. longipillus*–*P. koraiensis* system as the focus, this study found that *P. koraiensis* in the infested area exhibited systemic defense deficiencies: when attacked, it failed to effectively activate antioxidant responses and synthesize sufficient terpenes and other key defensive compounds, which may be the intrinsic reason for the extremely weak resistance of individual trees and the rapid collapse of their defense [33,34,35,36]. Like other secondary pests [37,38], this beetle preferentially attacked already weakened and damaged trees, and particularly concentrated on stress-prone Class III trees. However, the simultaneous presence of males and females significantly increased the host selection rate (Figure 2B,C), suggesting that at high population densities, they may gain the ability to mass-attack healthy trees through cooperative attacks, thereby posing a potential risk of transitioning from attacking weakened trees to eruptive spread [39]. Such a shift from secondary to primary pest behavior has also been observed in studies on *Ips typographus* [40,41]. Based on the general pattern that terpene volatiles released by damaged conifers can regulate bark beetle aggregation behavior [42,43], the active components of the attractant developed in this study, such as (+)-α-pinene and 3-carene, are also effective constituents of many bark beetle attractants [44,45]. Sweeney et al. [46] reported extremely low trap catches of *H. longipillus* in the Russian Far East using lures containing α-pinene, (-) β-pinene, (+)-limonene and α-terpinolene plus ethanol, further indicating that this beetle has historically not been a severe pest in this region. However, given that their study was conducted over a decade ago and climate change has increasingly triggered bark beetle outbreaks worldwide, previously minor species may pose greater risks in the future. In practice, an integrated control system was formed by combining the removal and destruction of infested trees, the trapping and killing of residual beetle populations in the forest, push–pull control using attractants and repellents, and the establishment of a long-term monitoring network, achieving remarkable effectiveness. This approach has also achieved significant success in the control of *I. typographus* [47]. Although chemical pesticides were applied to treat stumps during the emergency control process, the ecological and economic benefits recovered far outweighed the costs.

In forest stands where *H. longipillus* occurred, we successfully kept the population at a low level by elucidating its occurrence patterns and the chemical ecology mechanisms underlying its host selection, and by adopting targeted measures. Outbreaks of secondary bark beetles typically originate from the decline in tree vigor; their pioneer individuals carry symbiotic microorganisms that can accelerate host decline [48], while the specific volatiles released by weakened trees further guide the aggregation of conspecific individuals [49]. For example, previous studies have demonstrated that compounds released by stressed *Picea abies* provide attack cues for *I. typographus* [50,51]. This chemically mediated aggregation behavior allows bark beetles to reproduce rapidly within weakened trees [52,53]. The population grows exponentially, thereby gaining the potential to attack healthy trees [54]. The core of this control strategy lies in precisely severing the key link of this cycle: by removing weakened and infested trees and trapping and killing residual adults, the carrier and foundation for population accumulation are directly eradicated, forcing the bark beetles to return to a low-density state. Thereafter, should they attempt to rebound, they must repeat the entire process of “identifying and weakening hosts—aggregating and reproducing—spreading and causing damage,” a process that is subject to multiple uncertainties such as host spatial distribution and population base, making it difficult to achieve. Coupled with continuous monitoring, early outbreak signals of *H. longipillus* can be promptly detected and responded to, thereby nipping the risk of re-outbreak in the bud.

In multiple infested areas, relatively large populations of *H. longipillus* have become established, causing substantial mortality of *P. koraiensis* and highlighting the urgency of emergency control of *H. longipillus*. This emergency control operation strictly followed the IPM framework: first, infested trees with concentrated mortality were removed; then, a “push–pull” strategy centered on attractants and repellents was adopted [55], and intensive trapping and killing of the residual population was conducted. This control effort was promptly initiated at the early stage of the outbreak, effectively curbing further spread of the infestation, preventing larger-scale losses, and fully demonstrating the critical role of early intervention. Overall, the long-term management of *H. longipillus* should center on sustained monitoring, so as to achieve timely early warning and precise control of its population dynamics.

In recent years, bark beetle infestations have become a critical challenge in global forest pest management [56,57]. Particularly alarming is that the severe damage caused to *P. koraiensis* by *H. longipillus* signals a problem that may become increasingly prominent in the context of global climate change: some bark beetles not previously recorded as major threats may well transform into potent forest destroyers. Therefore, the management of such pests should be elevated to a higher level of priority. If targeted and effective control systems can be established, and highly efficient monitoring tools can be developed at the early stage of infestation, immeasurable ecological and economic losses may be prevented. Taking *H. longipillus* as an example, and given that its outbreak mechanisms remain unclear, this study explored and implemented an “emergency management paradigm based on rapid behavioral intervention.” This work not only provides specific theoretical insights and practical methods for the control of such sudden pest outbreaks, but also offers a transferable strategy for addressing the potentially increasing “atypical” and “sporadic” pest outbreaks in the future.

This study has certain limitations. First, the control protocol adopted was based on emergency response logic, and the formulated attractant still has room for improvement in specificity and long-lasting efficacy; thus, this protocol may not represent the optimal strategy for controlling *H. longipillus*. Second, the study was restricted to Shangzhi City, Heilongjiang Province, China; therefore, the occurrence and control of *H. longipillus* in other regions should be adjusted, or new methods should be developed based on local ecological conditions. More importantly, the specific driving factors of this outbreak (such as drought, disease, or other stresses) and their interaction network remain unclear, and long-term in-depth research is still needed. In addition, although some of the compounds used in this study were of relatively high purity grades, they inevitably contained unknown impurities. However, since the ultimate goal of this research is to support field pest management, all behavioral bioassays were conducted with practical application in mind. The overall consistency between our laboratory and field data indicates that impurities did not significantly interfere with our core conclusions, nor do they affect the evaluation of the overall efficacy of the attractant. However, this control practice demonstrates that successful emergency control does not depend on a complete understanding of all ecological mechanisms, but rather on precisely identifying and blocking the most critical and actionable link—in this study, the chemical communication process. This suggests that, when facing complex and sudden ecological disasters, the strategy of “rapidly identifying and targeting the most controllable link” is often more efficient than pursuing a comprehensive understanding of all mechanisms. In the future, while improving the monitoring system and control techniques for *H. longipillus*, the multi-factor driving mechanisms underlying its outbreaks should be further explored.

## 5. Conclusions

To address the outbreak of *H. longipillus*, rather than waiting for a complete understanding of its overall ecological mechanisms, this study rapidly identified its key chemical ecology vulnerability—namely, its dependence on specific volatiles and their isomers from *P. koraiensis*—and successfully developed a highly effective attractant. With this as the core, we integrated strategies including the removal and destruction of infested trees, the trapping and killing of residual populations, and the protection of healthy trees, thereby forming an integrated management plan, and effectively suppressed the outbreak in practice, validating the feasibility and effectiveness of this strategy. Therefore, this study not only deepened the understanding of this specific pest, but also established and validated a rapid response model for addressing emerging forest biosecurity threats. This model starts from systematic monitoring, rapidly diagnoses behavioral and chemical ecology mechanisms, develops targeted tools and integrates them into an integrated management system, and ultimately achieves effect evaluation, providing a complete actionable pathway for the management of similar sudden pest outbreaks.

## Figures and Tables

**Table 1:** Components of the formulated plant-derived semiochemicals for *H. longipillus*.

Formulation Type	Components	Mass Ratio
Attractant I	(+)-α-pinene, (-)-α-pinene, α-pinene, β-pinene, 3-carene, ethanol	7:7:2:2:2:20
Attractant II	(+)-α-pinene, (-)-α-pinene, α-pinene, β-pinene, 3-carene, ethanol	1:1:1:1:1:5
Repellent I	(+)-longifolene, bornyl acetate, (-)-limonene, ethanol	3:8:3:14
Repellent II	(+)-longifolene, bornyl acetate, (-)-limonene, ethanol	1:1:1:3
Control I	ethanol	
Control II	(+)-α-pinene, ethanol	1:1

## Data Availability

The data that support the findings of this study are available from the corresponding author upon reasonable request.

## References

[1] Inward D., Vuts J., Thomas G., Barnard K., Caulfield J.C., Powers S.J., Uglow A., Reed K. (2025). Investigating the threat to Sitka spruce from *Ips typographus*: Discrimination and colonization of Britain’s principal commercial conifer by a damaging forest pest. Pest Manag. Sci..

[2] Lecoq M. (1999). Deforestation, drought and exceptional outbreaks of the oriental migratory locust, *Locusta migratoria manilensis* (Meyen 1835) in Indonesia. J. Orthoptera Res..

[3] Ma K., Geng M., Han L., Sun Q., Zhang L., Yang Z., Tang Y., Guo S., Xiao Y., Zhang D. (2023). Carbon accounting of Weihe CSA pilot demonstration area in Longjiang forest industry. Processes.

[4] Hao J., Hou D., Yu W., Zhang H., Guo Q., Zhang H., Xiong H., Li Y. (2025). Metabolomic and transcriptomic analysis of the synthesis process of unsaturated fatty acids in Korean pine seed kernels. Food Chem..

[5] Yang Y., Jia Z., Ge S., Li Y., Kang D., Li J. (2025). Determining large trees and population structures of typical tree species in Northeast China. Diversity.

[6] Li X., Liu X.-T., Wei J.-T., Li Y., Tigabu M., Zhao X.-Y. (2020). Genetic improvement of *Pinus koraiensis* in China: Current situation and future prospects. Forests.

[7] Zhou H., Wang W., Chi D., Hu H., Yang W., Ji H., Song J., Yue Q. (2026). Spatiotemporal dynamics and driving factors of *Dioryctria* in China. Pest Manag. Sci..

[8] Wang H., Liu C., Yue F., Yan D.-H., Lu Q. (2022). Identification of ophiostomatalean fungi associated with *Tomicus pilifer* infesting *Pinus koraiensis* in Northeastern China. Front. Microbiol..

[9] Chen H., Tang M. (2014). Spatial and temporal dynamics of bark beetles in Chinese white pine in Qinling Mountains of Shaanxi Province, China. Environ. Entomol..

[10] Sommerfeld A., Rammer W., Heurich M., Hilmers T., Müller J., Seidl R. (2021). Do bark beetle outbreaks amplify or dampen future bark beetle disturbances in Central Europe?. J. Ecol..

[11] Valor T., Hood S.M., Piqué M., Larrañaga A., Casals P. (2021). Resin ducts and bark thickness influence pine resistance to bark beetles after prescribed fire. For. Ecol. Manag..

[12] Trubin A., Mezei P., Zabihi K., Surový P., Jakuš R. (2022). Northernmost European spruce bark beetle *Ips typographus* outbreak: Modelling tree mortality using remote sensing and climate data. For. Ecol. Manag..

[13] Selikhovkin A.V., Mamaev N.A., Martirova M.B., Merkuriev S.A., Popovichev B.G. (2022). A new outbreak of the European spruce bark beetle, *Ips typographus* (L.) (Coleoptera, Curculionidae) in Leningrad Province. Entomol. Rev..

[14] Cognato A.I., Sun J.-H., Anducho-Reyes M.A., Owen D.R. (2005). Genetic variation and origin of red turpentine beetle (*Dendroctonus valens* LeConte) introduced to the People’s Republic of China. Agric. For. Entomol..

[15] Kandasamy D., Gershenzon J., Hammerbacher A. (2016). Volatile organic compounds emitted by fungal associates of conifer bark beetles and their potential in bark beetle control. J. Chem. Ecol..

[16] Zhao T., Ganji S., Schiebe C., Bohman B., Weinstein P., Krokene P., Borg-Karlson A.-K., Unelius C.R. (2019). Convergent evolution of semiochemicals across kingdoms: Bark beetles and their fungal symbionts. ISME J..

[17] Ontiveros-Cisneros A., Salfeld J., Paulsson S., Ding B.-J., Wang H.-L., Friberg M., Löfstedt C., Van Aken O. (2025). A plant-based platform for the production of bark beetle pheromones. Plant Biotechnol. J..

[18] Chen H., Xie D., Pan Z., Jia N., Zhou J., Yang Q., Yu J., Chi D. (2025). Behavior-based stratified management: Population suppression and invasion front control of *Hylurgus ligniperda* (Fabricius) in coastal shelterbelt ecosystems. Pest Manag. Sci..

[19] Romeiro J.M.N., Strîmbu V.F., Eid T., Kangas A. (2025). Optimizing forest management in the face of bark beetle risk. Ecol. Model..

[20] Fares S., Mugnozza G.S., Corona P., Palahí M. (2015). Sustainability: Five steps for managing Europe’s forests. Nature.

[21] Lubojacký J., Holuša J. (2014). Attraction of *Ips typographus* (Coleoptera: Curculionidae) beetles by lure-baited insecticide-treated tripod trap logs and trap trees. Int. J. Pest Manag..

[22] Jakuš R. (1998). A method for the protection of spruce stands against *Ips typographus* by the use of barriers of pheromone traps in north-eastern Slovakia. Anz. Schädlingskd Pflanzenschutz Umweltschutz.

[23] Chakrabarty S., Deb C.K., Marwaha S., Haque M.A., Kamil D., Bheemanahalli R., Shashank P.R. (2026). Application of artificial intelligence in insect pest identification—A review. Artif. Intell. Agric..

[24] Göthlin E., Schroeder L.M., Lindelöw A. (2000). Attacks by *Ips typographus* and *Pityogenes chalcographus* on windthrown spruces (*Picea abies*) during the two years following a storm felling. Scand. J. For. Res..

[25] Fettig C.J., Klepzig K.D., Billings R.F., Munson A.S., Nebeker T.E., Negrón J.F., Nowak J.T. (2007). The effectiveness of vegetation management practices for prevention and control of bark beetle infestations in coniferous forests of the western and southern United States. For. Ecol. Manag..

[26] Gillette N.E., Wood D.L., Hines S.J., Runyon J.B., Negrón J.F. (2014). The once and future forest: Consequences of mountain pine beetle treatment decisions. For. Sci..

[27] Cook S.M., Khan Z.R., Pickett J.A. (2007). The use of push-pull strategies in integrated pest management. Annu. Rev. Entomol..

[28] Ramakrishnan R., Shewale M.K., Strádal J., Frühbrodt T., Doležal P., Hani U.E., Andersson M.N., Gershenzon J., Jirošová A. (2025). Aggregation pheromones in the bark beetle genus *Ips*: Advances in biosynthesis, sensory detection, and forest management applications. Curr. For. Rep..

[29] Lantschner M.V., Corley J.C. (2023). Spatiotemporal outbreak dynamics of bark and wood-boring insects. Curr. Opin. Insect Sci..

[30] Gao L., Li Y., Wang Z.-X., Zhao J., Hulcr J., Wang J.-G., Li Y.-Z., Ju R.-T. (2021). Biology and associated fungi of an emerging bark beetle pest, the sweetgum inscriber *Acanthotomicus suncei* (Coleoptera: Curculionidae). J. Appl. Entomol..

[31] Jaime L., Batllori E., Lloret F. (2024). Bark beetle outbreaks in coniferous forests: A review of climate change effects. Eur. J. For. Res..

[32] Jian S., Han Y., Kasanen R., Honkaniemi J., Junttila S., Asiegbu F.O. (2025). Implications for the distributional range of the European bark beetles under future climate change. Sci. Rep..

[33] Jena K., Kar P.K., Kausar Z., Babu C.S. (2013). Effects of temperature on modulation of oxidative stress and antioxidant defenses in testes of tropical tasar silkworm Antheraea mylitta. J. Therm. Biol..

[34] Mithöfer A., Boland W. (2012). Plant defense against herbivores: Chemical aspects. Annu. Rev. Plant Biol..

[35] Kolosova N., Bohlmann J., Matyssek R., Schnyder H., Oßwald W., Ernst D., Munch J.C., Pretzsch H. (2012). Conifer defense against insects and fungal pathogens. Growth and Defence in Plants: Resource Allocation at Multiple Scales.

[36] Franceschi V.R., Krokene P., Christiansen E., Krekling T. (2005). Anatomical and chemical defenses of conifer bark against bark beetles and other pests. New Phytol..

[37] Ray C., Cluck D.R., Wilkerson R.L., Siegel R.B., White A.M., Tarbill G.L., Sawyer S.C., Howell C.A. (2019). Patterns of woodboring beetle activity following fires and bark beetle outbreaks in montane forests of California, USA. Fire Ecol..

[38] Pirtskhalava-Karpova N., Trubin A., Karpov A., Jakuš R. (2024). Drought initiated bark beetle outbreak in Central Europe: Meteorological factors and infestation dynamic. For. Ecol. Manag..

[39] Chiu C.C., Bohlmann J. (2022). Mountain pine beetle epidemic: An interplay of terpenoids in host defense and insect pheromones. Annu. Rev. Plant Biol..

[40] Lieutier F., Day K., Battisti A., Grégoire J.-C., Evans H. (2004). Bark and Wood Boring Insects in Living Trees in Europe, a Synthesis.

[41] Kausrud K., Okland B., Skarpaas O., Grégoire J.C., Erbilgin N., Stenseth N.C. (2012). Population dynamics in changing environments: The case of an eruptive forest pest species. Biol. Rev..

[42] Gallego D., Galián J., Diez J.J., Pajares J.A. (2008). Kairomonal responses of *Tomicus destruens* (Col., Scolytidae) to host volatiles α-pinene and ethanol. J. Appl. Entomol..

[43] Shi X., Fang J., Du H., Zhang S., Liu F., Zhang Z., Kong X. (2022). Performance of two *Ips* bark beetles and their associated pathogenic fungi on hosts reflects a species-specific association in the beetle-fungus complex. Front. Plant Sci..

[44] Erbilgin N., Raffa K.F. (2000). Opposing effects of host monoterpenes on responses by two sympatric species of bark beetles to their aggregation pheromones. J. Chem. Ecol..

[45] Lu M., Wingfield M.J., Gillette N.E., Mori S.R., Sun J.-H. (2010). Complex interactions among host pines and fungi vectored by an invasive bark beetle. New Phytol..

[46] Sweeney J.D., Silk P., Grebennikov V., Mandelshtam M. (2016). Efficacy of semiochemical-baited traps for detection of Scolytinae species (Coleoptera: Curculionidae) in the Russian Far East. Eur. J. Entomol..

[47] Wermelinger B. (2004). Ecology and management of the spruce bark beetle *Ips typographus*—A review of recent research. For. Ecol. Manag..

[48] Lu M., Hulcr J., Sun J. (2016). The role of symbiotic microbes in insect invasions. Annu. Rev. Ecol. Evol. Syst..

[49] Bruce T.J., Wadhams L.J., Woodcock C.M. (2005). Insect host location: A volatile situation. Trends Plant Sci..

[50] Lehmanski L.M., Kandasamy D., Andersson M.N., Netherer S., Alves E.G., Huang J., Hartmann H. (2023). Addressing a century—Old hypothesis—Do pioneer beetles of *Ips typographus* use volatile cues to find suitable host trees?. New Phytol..

[51] Schiebe C., Hammerbacher A., Birgersson G., Witzell J., Brodelius P.E., Gershenzon J., Hansson B.S., Krokene P., Schlyter F. (2012). Inducibility of chemical defenses in Norway spruce bark is correlated with unsuccessful mass attacks by the spruce bark beetle. Oecologia.

[52] Cano-Ramírez C., Armendáriz-Toledano F., Macías-Sámano J.E., Sullivan B.T., Zúñiga G. (2012). Electrophysiological and behavioral responses of the bark beetle *Dendroctonus rhizophagus* to volatiles from host pines and conspecifics. J. Chem. Ecol..

[53] Vité J.P., Pitman G.B. (1968). Bark beetle aggregation: Effects of feeding on the release of pheromones in *Dendroctonus* and *Ips*. Nature.

[54] Raffa K.F., Aukema B.H., Bentz B.J., Carroll A.L., Hicke J.A., Turner M.G., Romme W.H. (2008). Cross-scale drivers of natural disturbances prone to anthropogenic amplification: The dynamics of bark beetle eruptions. BioScience.

[55] Gillette N.E., Mehmel C.J., Mori S.R., Webster J.N., Wood D.L., Erbilgin N., Owen D.R. (2012). The push-pull tactic for mitigation of mountain pine beetle (Coleoptera: Curculionidae) damage in lodgepole and whitebark pines. Environ. Entomol..

[56] Marini L., Økland B., Jönsson A.M., Bentz B., Carroll A., Forster B., Grégoire J.-C., Hurling R., Nageleisen L.M., Netherer S. (2017). Climate drivers of bark beetle outbreak dynamics in Norway spruce forests. Ecography.

[57] Marini L., Ayres M.P., Battisti A., Faccoli M. (2012). Climate affects severity and altitudinal distribution of outbreaks in an eruptive bark beetle. Clim. Change.

